# New Insight into CO_2_ Reduction to Formate by In Situ Hydrogen Produced from Hydrothermal Reactions with Iron

**DOI:** 10.3390/molecules27217371

**Published:** 2022-10-29

**Authors:** Xu Zeng, Guo-Dong Yin, Yang-Yuan Zhou, Jian-Fu Zhao

**Affiliations:** State Key Laboratory of Pollution Control and Resources Reuse, College of Environmental Science and Engineering, Tongji University, 1239 Siping Road, Shanghai 200092, China

**Keywords:** CO_2_, in situ reduction, hydrothermal reactions, formate

## Abstract

To reveal the nature of CO_2_ reduction to formate with high efficiency by in situ hydrogen produced from hydrothermal reactions with iron, DFT calculations were used. A reaction pathway was proposed in which the formate was produced through the key intermediate species, namely iron hydride, produced in situ in the process of hydrogen gas production. In the in situ hydrogenation of CO_2_, the charge of H in the iron hydride was −0.135, and the Fe–H bond distance was approximately 1.537 Å. A C-H bond was formed as a transition state during the attack of H^δ−^ on C^δ+^. Finally, a HCOO species was formed. The distance of the C-H bond was 1.107 Å. The calculated free energy barrier was 16.43 kcal/mol. This study may provide new insight into CO_2_ reduction to formate in hydrothermal reactions with metal.

## 1. Introduction

Global temperature has risen noticeably during the past few decades, mainly due to CO_2_ emissions [1]. As a result, many negative effects climate change affairs have been felt, which has caused a severe threat to human survival. Fortunately, many efforts have been made to reduce CO_2_ emissions. For example, the transformation of CO_2_ into chemicals or fuels has received much attention, which would not only reduce CO_2_ emissions but also alleviate the shortage of fossil fuels [2]. Therefore, establishing the most commercially feasible method for the hydrogenation of CO_2_ has become highly desirable [3]. However, the high kinetic and thermodynamic stability of CO_2_ hindered the reduction of CO_2_ emissions. Solar technologies are considered to be the ideal solutions to the greenhouse effect problem. However, the utilization of solar energy normally has the disadvantages of low efficiency and low production selectivity. On the other hand, the hydrogenation of CO_2_ with gaseous hydrogen is regarded as the most commercially feasible option. However, the gaseous hydrogen process is expensive due to the costs of production, storage, and transportation. Therefore, once the in situ produced hydrogen can be used for the hydrogenation of CO_2_, the problem of hydrogen storage and transportation can be solved easily. Thus, establishing an efficient CO_2_ conversion process is a necessary and fascinating challenge [4,5].

Hydrothermal reactions play an important role in the formation of fossil fuels, due to their special characteristics such as their low dielectric constant and high ion product [6]. Water under high temperature and high pressure can be used as a reaction medium and hydrogen source. The production of formate from CO_2_ reduction under hydrothermal conditions has been reported extensively [7,8,9]. Recently, a two-step CO_2_ reduction process based on water-splitting hydrogen production with the redox cycle of metals/metal oxides has been reported (for example, in relation to Fe/Fe_3_O_4_) [10]. In this process, the iron was used as a reductant, and the iron oxides were used to reproduce iron using solar energy, which is a kind of renewable energy. The hydrogen production efficiency from the dissociation of water under hydrothermal conditions was much higher than that with solar energy [11]. Thus, hydrogen production from hydrothermal reactions was assumed to be a sustainable method for the reduction of CO_2_. Consequently, the dissociation of water with metal under hydrothermal conditions would represent one of the most promising approaches to decrease CO_2_ emissions. Obviously, the conversion of CO_2_ to chemicals or fuels is the key step in this process. However, the hydrogenation of CO_2_ in hydrothermal reactions has not been studied in sufficient detail.

In recent years, we have studied extensively the metal hydrothermal reaction for CO_2_ reduction. Zinc hydride (Zn–H) is reported as a key intermediate species during the production of formate. The formate was produced through an SN2-like reaction [12]. In an experimental study, Zn–H species, produced from the reaction of Zn and H_2_O, were detected by using FT-IR and XPS analysis. In a theoretical study, the Zn–H species was detected as a key intermediate by using the quantum chemical calculations method. The reaction pathway detection can be used to explain the production of formate with Zn in hydrothermal reactions.

In our previous experimental study, we investigated the dissociation of H_2_O for reducing CO_2_ (or NaHCO_3_ as a CO_2_ source) to formic acid with metallic iron [13]. In this process, H_2_O is used as a source of hydrogen and Fe is used as a reductant, which can rapidly produce hydrogen under hydrothermal conditions. With the in situ produced hydrogen, CO_2_ was reduced to formic acid efficiently. After the reaction, Fe was oxidized to Fe_3_O_4_. It was also reported that NaHCO_3_ was reduced into formate with high efficiency by using Fe as a reductant [14]. The overall equation can be shown as:

$$3\mathrm{Fe}+{\mathrm{NaHCO}}_{3}+3H_{2}O\to {\mathrm{Fe}}_{3}O_{4}+3H_{2}(g)+\mathrm{HCOONa}$$

$$\Delta G\left(573\;K\right)=-132.776\;\mathrm{kJ}\cdot {\mathrm{mol}}^{-1}$$

In this study, a theoretical study was conducted by using the DFT calculations. Under hydrothermal reaction conditions, the in situ technique for experimentally studying the reaction mechanism of formate production is very hard to use. However, the ab initio calculation is very useful for studying the mechanism including reliable structures as well as the energies of the reactants, products, intermediates, and transition states.

## 2. Computational Details

DFT calculations, including a consideration of van der Waals (VDW) interactions, were adopted by using Gaussian 16 [15]. The B3LYP density functional method [16] with the D3 (GJ) dispersion correction was employed to carry out all of the computations. An SDD basis set was used for Fe atoms and a 6-31G+G(d,p) basis set was used for remaining atoms. Polarizable continuum model (PCM) calculations of water as the solvent were used in this work. Vibrational frequency analyses were conducted at the same level of theory to characterize stationary points as local minima. Intrinsic reaction coordinate (IRC) computations were carried out to confirm the transition states.

## 3. Results and Discussion

### 3.1. Analysis of Geometric Parameters

**Figure 1:**
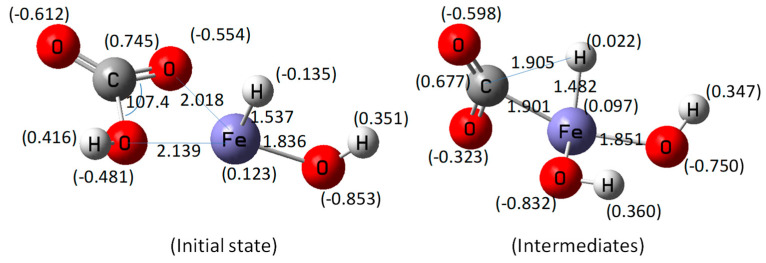
Optimized geometric parameters of the initial state and the intermediate state.

The geometric parameters of initial state and intermediate states are shown in Figure 1. Bond lengths are in Å and bond angles are in degrees. As shown in Figure 1, in the initial state, the bond distance of Fe–H was approximately 1.537 Å, and the Mulliken charge of H in Fe–H was −0.135. This resulting charge means that a hydride was formed, not a proton. The geometry and Mulliken charge distribution implied that the Fe–H species was similar to our previous study with zinc. Since metal hydride has high catalytic activity, it could be used as a reduction agent. The Fe–O bond distances between Fe and HCO_3_^-^ was approximately 2.0~2.1 Å. When the intermediate state is formed, the Fe–H bond distance was about 1.482 Å. The Mulliken charge of H in Fe–H was 0.022. The C–H bond distance was about 1.905 Å. In the initial state, the Fe–H^δ−^ species was formed via the dissociation of water. Based on the calculation results, it was assumed that the CO_2_ was produced from bicarbonate under hydrothermal conditions as an intermediate. Then, the intermediate state was used for the calculation for the formate production.

**Figure 2:**
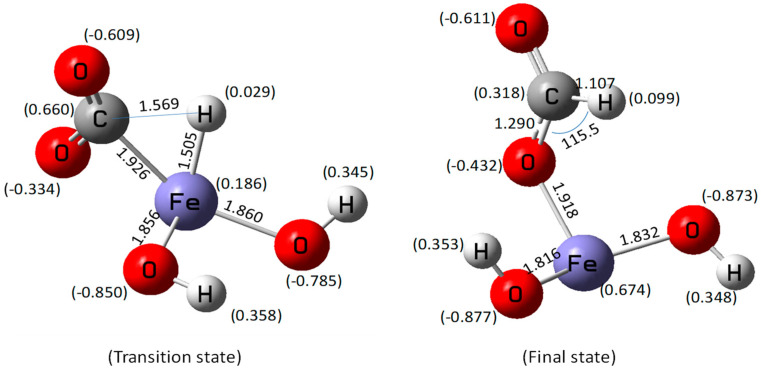
Optimized geometric parameters of the transition state and the final state.

The geometric parameters of the transition state and the final state are shown in Figure 2. The Fe–H bond distance in the transition state was approximately 1.505 Å, and the Mulliken charge of H in the iron hydride species was 0.029, as shown in Figure 2. The distance between C and H atoms was 1.569 Å. By comparing them with the distance in the intermediate state, one can see that the distance is closer. The distance between metal and H atoms is similar to our previous study on CO_2_ reduction by zinc, which was 1.490 Å. The Fe–O bond distance was approximately 1.85~1.86 Å. In the final state, the bond distance of C–H was 1.107 Å. The Mulliken charge of H in the C–H species was 0.099. Compared with the distance in the intermediate and the transition state, the distance of C–H bond was closer, at only 1.107 Å. It means that the C-H bond is stronger, and the final state is more stable. The Fe–O bond distances were approximately 1.8~1.9 Å, and the charge of Fe was 0.674. The bond angle between the H–C–O bond was 115.5°. Based on these bond distance results, we proposed the mechanism of HCOO production as follows. In the hydrogen production process, the Fe–H^δ−^ species was formed via the dissociation of water. Due to the high reducibility of H^δ−^, the distance between H and C atoms became closer. When a transition state during the attack of H^δ−^ on C^δ+^ formed, the HCOO species could be produced subsequently.

### 3.2. Energy Diagram for HCOO Production

**Figure 3:**
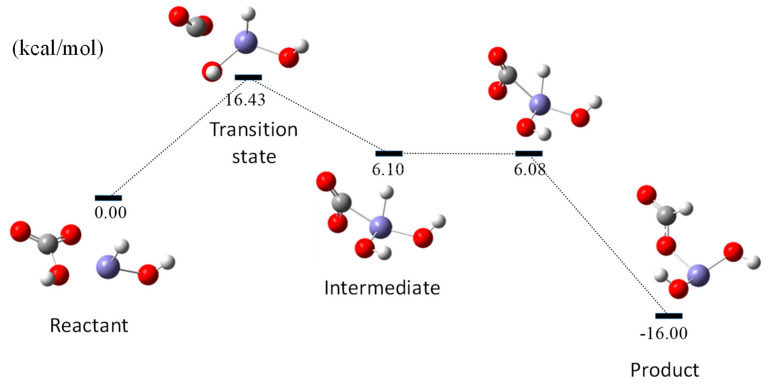
Potential energy diagram for HCOO production.

An energy diagram of the C–H bond formation is shown in Figure 3. The optimized geometry of the initial, intermediate, and final states is depicted in Figure 1 and Figure 2. As shown in Figure 3, the calculated activation energy is 16.43 kcal/mol. Compared with our previous study, when HCO_3_^−^ was used as the initial state, the calculated activation energy was 24.1 kcal/mol, which means that the CO_2_ was easier to reduce than HCO_3_^−^ under reaction conditions. An important implication of the calculated activation energy is that the in situ produced Fe–H species has high activity regarding reduction properties.

### 3.3. IRC and HOMO/LUMO Calculation of Transition State

**Figure 4:**
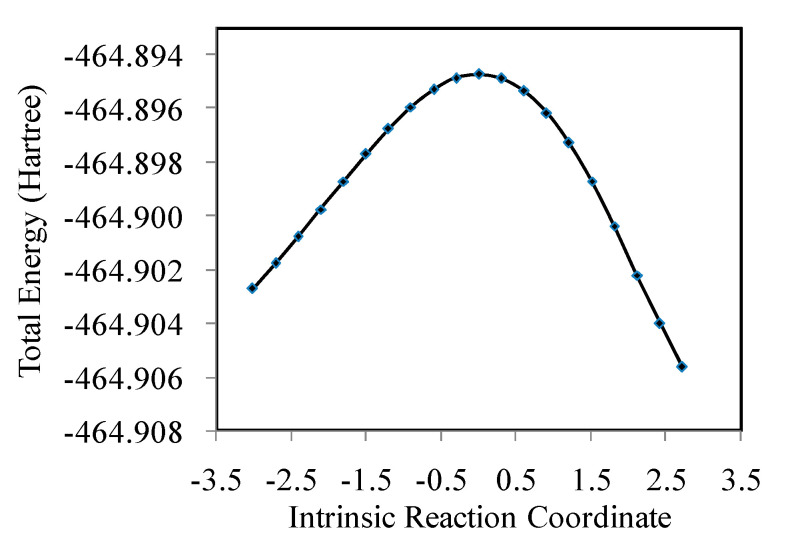
Intrinsic reaction coordinate calculation results.

**Figure 5:**
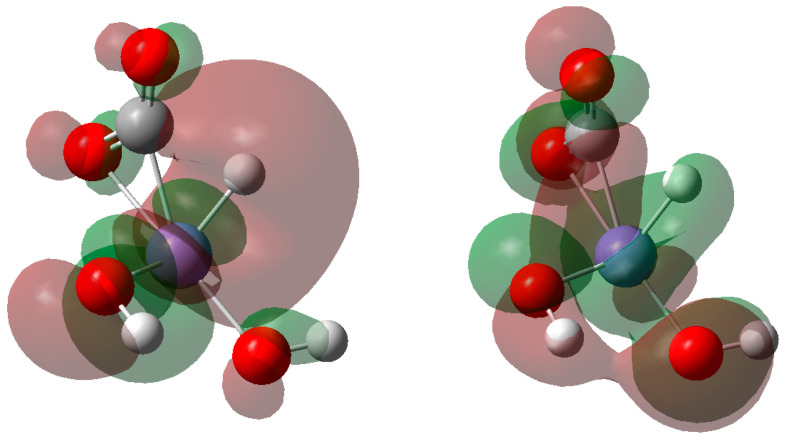
HOMO and LUMO orbital shapes of the transition state.

The IRC calculations and the HOMO and LUMO orbital shapes of the transition state (TS) are shown in Figure 4 and Figure 5, respectively. The IRC curve is smooth, as shown in Figure 4. The formate was produced in the vertex of the curve. It can easily be seen that the the reaction energy barrier was not very high, which means that formate could easily produced once the Fe–H species was formed. As shown in Figure 5, the occupied HOMO of the transition state exhibits a bonding interaction between the C atom and the H atom of the iron hydride species. On the other hand, the unoccupied LUMO of the transition state shows an anti-bonding character. These results clearly show the formation of a C–H bond of the formate.

## 4. Conclusions

In this study, CO_2_ reduction to formate by in situ hydrogen produced from hydrothermal reactions with iron was investigated through a theoretical study to provide new insights. A possible reaction pathway was proposed in which iron hydride, produced in situ under hydrothermal conditions, was a key intermediate species in formate production. The structures and energies were obtained, and the IRC and HOMO/LUMO orbit shapes were built using the 6-31G+G(d,p) basis set. In the hydrogen production process, the Fe–H^δ−^ species was formed via the dissociation of water. In the in situ hydrogenation process of CO_2_, the Mulliken charge of H in the iron hydride was −0.135, and the Fe–H bond distance was approximately 1.537 Å. A C–H bond was formed as a transition state during the attack of H^δ−^ on C^δ+^. Finally, a HCOO species was formed. The distance of the C-H bond was 1.107 Å. The calculated free energy barrier was 16.43 kcal/mol. These results showed that the reaction could take place with a lower formation barrier. This study may provide new insight into CO_2_ reduction to formate in hydrothermal reactions with metal, which will promote new ideas in the field of CO_2_ utilization.

## Data Availability

The data presented in this study are available on request from the corresponding author.
